# Development of a national root cause analysis system for post-endoscopy upper gastrointestinal cancer

**DOI:** 10.1055/a-2788-3285

**Published:** 2026-03-16

**Authors:** Amar Srinivasa, Rebeca Fiadeiro, Tim Gentry, Shilpi Goel, Tameera Rahman, Karen Clements, Warren Chapman, Nicholas Burr, Dipankar Mukherjee, Matthew Banks, Mimi McCord, Anjan Dhar, Roland Valori, Nigel Trudgill

**Affiliations:** 1 1731 Gastroenterology, Sandwell and West Birmingham NHS Trust, West Bromwich, United Kingdom of Great Britain and Northern Ireland; 2 1724 Institute of applied health research, University of Birmingham, Birmingham, United Kingdom of Great Britain and Northern Ireland; 3 Health Data Insight CIC, Cambridge, United Kingdom of Great Britain and Northern Ireland; 4 496847 National Disease Registration Service, London, United Kingdom of Great Britain and Northern Ireland; 5 Cancer Epidemiology Group, Institute of Cancer & Pathology and Institute of Data Analytics, Leeds University, Leeds, United Kingdom of Great Britain and Northern Ireland; 6 6965 Barking Havering and Redbridge Hospitals NHS Trust, Romford, United Kingdom of Great Britain and Northern Ireland; 7 8964 Department of Gastroenterology, University College London Hospitals NHS Foundation Trust, London, United Kingdom of Great Britain and Northern Ireland; 8 Heartburn Cancer UK, Heartburn Cancer UK, Chichester, United Kingdom of Great Britain and Northern Ireland; 9 3058 County Durham and Darlington NHS Foundation Trust, Darlington, United Kingdom of Great Britain and Northern Ireland; 10 Gloucestershire Hospitals NHS Trust, Gloucester, United Kingdom of Great Britain and Northern Ireland; 11 1724 Department of Cancer and Genomic Sciences, University of Birmingham, Birmingham, United Kingdom of Great Britain and Northern Ireland

**Keywords:** Endoscopy Upper GI Tract, Quality and logistical aspects, Quality management, Performance and complications

## Abstract

**Background and study aims:**

Post-endoscopy upper gastrointestinal cancer (PEUGIC) is a key performance indicator for endoscopy. PEUGIC represents a delay in diagnosis: a patient has an endoscopy that did not diagnose cancer, and another investigation, usually endoscopy, 3 to 36 months later that diagnoses upper gastrointestinal (UGI) cancer. We describe a national system to identify PEUGIC and undertake root cause analysis.

**Methods:**

This retrospective national study was undertaken in the English National Health Service (NHS) and consisted of: 1) identification of PEUGIC; 2) development of an online platform for root cause analysis; and 3) pooled national analysis of PEUGIC. Two national datasets--Hospital Episode Statistics and National Cancer Registration and Analysis Service’s cancer registry--enabled identification of people diagnosed with UGI cancer who had endoscopy in the previous 3 to 36 months without a cancer diagnosis (PEUGIC). The online portal informed every endoscopy provider of their PEUGIC and enabled a comprehensive root cause analysis.

**Results:**

This methodology was successful in identifying 3907 PEUGIC from January 1, 2017 to October 30, 2023. Root cause analysis was completed for 2666 PEUGIC during the study period and represents the world’s largest cohort of PEUGIC. 664 (17%) PEUGIC were ineligible on local review as they did not meet study criteria.

**Conclusions:**

A process to identify PEUGIC across a national healthcare system and to perform root cause analysis is described. The methodology is transferable to other healthcare systems with large national datasets, but even without such datasets, the root cause analysis process developed allows identification of learning from PEUGIC for local endoscopy quality improvement.

## Introduction

Approximately 15,900 people in the United Kingdom are diagnosed with esophageal or stomach cancer annually. Unfortunately, the outlook for people with these cancers is often poor, with only 16% surviving esophageal and 19% surviving gastric cancer for 5 years 1 2 3 . There are a number of potential reasons for these disappointing outcomes, but one important factor is the quality of upper gastrointestinal (UGI) endoscopy.

Over 1,100,000 UGI endoscopies are undertaken each year in the UK 4 , but endoscopy is not infallible and sometimes cancer, or a lesion that will later become malignant, is not found. Approximately 9% of patients diagnosed with UGI cancers had an endoscopy in the previous 3 years that did not diagnose their cancer 5 6 7 8 . These cancers are known as post-endoscopy upper gastrointestinal cancer (PEUGIC). In 42% of these patients, their treatment was adversely affected and 9% died prematurely due to PEUGIC 9 .

In England, some hospitals seem to miss more cancers than others, with a three-fold variation in PEUGIC rates between hospitals 10 . However, studies examining risk factors for PEUGIC and post-colonoscopy colorectal cancer (PCCRC), have predominantly been population-based and lack the granular detail needed for targeted interventions to reduce their occurrence 7 11 12 13 14 15 . Root cause analysis provides the reasons why adverse events (AEs) such as PEUGIC occur and following the identification of contributory factors, allows interventions to reduce their occurrence 16 .

For an individual endoscopy provider, as a relatively rare event, identifying PEUGIC can be challenging 5 . National studies have also shown that 20% of PEUGIC are diagnosed at a different provider than the non-diagnostic (index) endoscopy provider, so the PEUGIC would not usually be known to the index endoscopy provider 10 . Therefore, a national process is required to identify PEUGIC with a standardized root cause analysis process to allow analysis of data on PEUGIC locally and identification of contributory factors for PEUGIC, leading to interventions to reduce their occurrence.

## Patients and methods

### Study design

The national PEUGIC root cause analysis project was a multicenter, retrospective cohort study to identify underlying contributory factors to PEUGIC. The study involved 144 endoscopy providers in the National Health Service (NHS) in England. The study had three main aims: 1) identification of PEUGIC across the English NHS; 2) development of a secure web-based portal for endoscopy providers to perform local root cause analysis of each PEUGIC; and 3) analysis of pooled findings nationally to describe the causes of and interventions for PEUGIC. Endoscopy providers were eligible if they were part of the English NHS. Independent sector endoscopy providers were excluded because their data were not included in the national datasets used for the project.

### Study definition of PEUGIC

At the time of the study, there was no internationally agreed definition for PEUGIC. However, a number of studies on PEUGIC have proposed a definition in their inclusion criteria 5 6 7 17 18 19 20 21 22 23 24 25 26 27 28 29 30 . PEUGIC was defined as a diagnosis of UGI (esophageal, gastric or duodenal) cancer that occurred following an index endoscopy that did not diagnose cancer. There was a need to define a time period following index endoscopy for study inclusion 31 . At the time of study design, most studies on PEUGIC used 36 months as the upper time limit for PEUGIC 5 6 7 17 18 19 20 21 22 23 24 25 26 27 29 30 . Moreover, there is significant variation in the starting time point with studies choosing 3 months 5 6 28 29 or 6 months 7 17 24 25 post index endoscopy. In previous unpublished work by the study team, we noted that when a PEUGIC interval of 3 to 36 months was used, 9.0% of PEUGICs were diagnosed between 3 to 6 months after the index endoscopy. Given this large number of PEUGIC and a lack of data on PEUGIC during this time period and how preventable they were, it was decided that root cause analysis of these PEUGIC would be of benefit and 3 months post index endoscopy, therefore, was chosen as the lower time limit.

### PEUGIC ascertainment

**Fig. 1:**
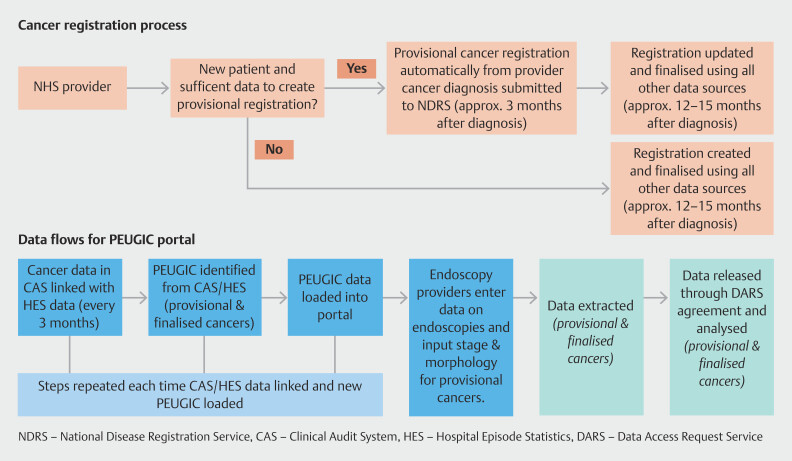
Data flows for the national PEUGIC root cause analysis project.

PEUGIC were identified using linkage of two national datasets. The National Cancer Registration and Analysis Service (NCRAS) cancer registration dataset held by the National Disease Registry Service (NDRS) provides information on cancer site, staging, histology, and date of diagnosis. Both final and provisional cancer registrations were used to provide the most contemporaneous data, to be most relevant to provider current practice. The Hospital Episode Statistics (HES) dataset provides information on endoscopy, including the date and which provider carried out the endoscopy. Fig. 1 summarizes data flows in the project. Linkage between the two datasets allows identification of PEUGIC using International Classification of Diseases (ICD-10) codes in the cancer registry linked to Office of Population Censuses and Surveys (OPCS-4) codes in HES (code list in Supplementary Table 1, Supplementary Table 2, and Supplementary Table 3). Patients under the age of 18 were excluded from the case ascertainment phase.

### Recruitment of endoscopy providers in England

A total of 144 NHS endoscopy providers in England with at least 10 PEUGICs in the past 5 years were identified.

Providers were contacted to nominate a PEUGIC project lead for the organization. PEUGIC project leads were the point of contact for the PEUGIC project team and were responsible for participation in the project. Oversight by a consultant (senior independent specialist doctor) was required to assume responsibility for any duty of candor issues that could arise.

PEUGIC leads were asked to register the project with their clinical effectiveness/quality improvement/audit department and to inform endoscopist colleagues about participation in the project. An information governance guidance document, a data protection impact assessment approved by the NDRS Caldicott Guardian, a project summary, and a letter for endoscopist colleagues were provided.

### Development of root cause analysis questionnaire

A root cause analysis questionnaire was developed by the project team based on the previous PCCRC audit and PEUGIC studies. This included sections about the cancer and the index endoscopy. The main root cause analysis questions are summarized in Table 1 .

**Table:** **Table 1** Root cause analysis questionnaire items.

Cancer questions	Index endoscopy questions
Predisposing risk factors for upper gastrointestinal cancer (eg, Barrett’s esophagus, chronic atrophic gastritis, gastric intestinal metaplasia, high-risk cohorts such as chronic liver disease, chronic obstructive pulmonary disease or previous head and neck or lung cancer) Cancer site (e.g. esophagus, gastro-esophageal junction Siewert I/II/II, stomach or duodenum) Cancer histology Cancer differentiation Cancer staging (TNM and overall stage) Test that led to cancer diagnosis Cancer size (horizontal or width in mm) Macroscopic appearance (e.g. exophytic, ulcerated, or diffusely infiltrating) Cancer treatment intent Cancer treatment received If resected: Microscopic tumor extension (pT) Number of regional lymph nodes evaluated/number of positive lymph nodes (pN) Vascular or lymphatic invasion Perineural invasion Perineural invasion Resection margins Pathological staging (TNM) Impact of delay in diagnosis for the patient (e.g. potential endoscopic resection instead of surgery or potential surgery instead of palliative care) if diagnosed at index endoscopy PEUGIC avoidable (e.g. no, possibly, probably or definitely) Patient harmed (e.g. mild, moderate, major or premature death due to PEUGIC)	Date and time of index endoscopy Procedure type (inpatient, outpatient or therapeutic) Procedure indication (screening, surveillance, symptomatic, abnormal imaging or other) Urgency of procedure (emergency, urgent-cancer pathway, urgent non-cancer pathway or routine) Endoscopist role/speciality Training episode Route of intubation Combined with lower gastrointestinal endoscopy Endoscope manufacturer Endoscope series Premedication before endoscopy Degree of sedation Mucosal cleansing used during endoscopy Image enhancement used during endoscopy Evidence from the endoscopy report of poor tolerance Quality of mucosal view Extent of examination (including photodocumentation of any lesion sampled and when extent allows photodocumentation of retroflexion in fundus and D2) Location and photodocumentation of all visualized cancer associated lesions and pre-malignant lesions Reason for incomplete endoscopy Location and number of biopsies taken Estimated size of all visualized cancer associated lesions Prague classification of visualized segments of Barrett’s esophagus Description of focal lesions Endoscopy report indicates a difficult or lengthy procedure Endoscopy recognized by endoscopist to be inadequate and reason Person/team responsible for follow-up plan Repeat or alternative test or endoscopic therapy Non-procedural factors contributing to PEUGIC: Patient factors (e.g. patient did not attend follow-up or declined further investigation), Administrative factors (e.g. booking delay), Clinical decision making factors (e.g. inappropriate decision, decision not acted upon) PEUGIC categorization and subtype
PEUGIC, Post Endoscopy Upper Gastrointestinal cancer.	

In addition, an eligibility check was placed at the start of the root cause analysis questionnaire, which helped reviewers determine patient eligibility for the project. Demographic details about the patient, diagnosis date, cancer site, morphology, and endoscopy date required confirmation, along with confirmation for duodenal cancers that the cancer was within the first or second part of the duodenum, so that the PEUGIC was in the region normally examined during UGI endoscopy.

The root cause analysis questionnaire was tested by 13 pilot endoscopy providers and refined following feedback prior to national roll out. This also allowed the online portal to be tested for errors prior to national rollout.

### Development of a root cause analysis portal

The PEUGIC root cause analysis portal was added to the NDRS Clinical Audit System. The Clinical Audit System is a secure website which can only be accessed on the Health and Social Care Network, a data network for health and care organizations that is maintained by NHS England.

The Clinical Audit System homepage contains a link to a registration page. To register, users are requested to provide their name, NHS (or NHS accredited) email address, mobile phone number for two factor authentication, General Medical Council or Nursing and Midwifery Council membership numbers, and to select which organization(s) from the list of identified NHS endoscopy providers they would like to register for.

The PEUGIC project team created accounts based on this information and triggered a secure email with log-in details. Names were confirmed using the General Medical Council or Nursing and Midwifery Council registers. Each new account had to be validated by the organization project lead through the portal before new users were allowed access.

Once logged in, users had access to the following pages: 1) Manage portal users - exclusive to organization leads to add, approve or remove other users from their organization; 2) Manage PEUGIC cases - a dashboard with identified PEUGIC for the registered organization and a link to review the PEUGIC using the root cause analysis questionnaire; 3) Contact us page - an opportunity to send a message, which was securely sent to the project team NHS e-mail inbox; and 4) Documentation page - a collection of guidance documents created for the project.

### Guidance documents

To facilitate standardized root cause analysis, a number of guidance documents were created to support reviewers, particularly when there was no prior guidance in areas such as PEUGIC categorization, definition of adequate endoscopy, determination of harm, avoidability, and duty of candor. Local project leads were advised to discuss PEUGIC in a suitable governance forum with other endoscopists when there was significant harm to the patient and the PEUGIC was at least probably avoidable, to establish local consensus concerning discharging the duty of candor 32 .

**Fig. 2:**
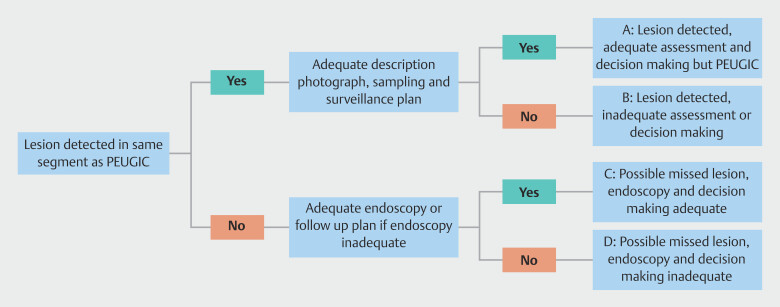
PEUGIC categorization and examples. A: Patient undergoing Barrett’s surveillance is found to have a nodule in their segment of Barrett’s. Biopsy shows high-grade dysplasia and the patient has an endoscopic recection after 4 months and is found to have intramucosal cancer. B: Patient found to have a gastric ulcer but not biopsied due to concern over risk of bleeding. Repeat endoscopy delayed and at 12 months confirms gastric cancer. C: Patient has an endoscopy for dyphagia and found to have no abnormalities. Presents again with worsening dysphagia 20 months later and found to have established esophageal squamous cell cancer. D: Patient has an endoscopy for persistent vomiting and this is abandoned due to food in the stomach. Barium meal suggests gastroparesis. Patient represents 12 months later with gastric outlet obstruction secondary to gastric cancer.

Categorization of preventability was adapted from the World Endoscopy Organization’s PCCRC consensus document and Kamran et al. 2022 31 33 . PEUGIC categorization is summarized in Fig. 2 and was a two-step process involving an initial assessment of whether a lesion was noted at the index endoscopy at the site of the later cancer and then an assessment of adequacy of endoscopy and subsequent clinical care, including follow-up or surveillance decisions. Categorization of PEUGIC is one of the most important steps in the root cause analysis process, highlighting which PEUGIC are likely to be preventable (categories B+D) and those that are only possibly preventable (categories A+C). Fig. 2 also provides examples and their PEUGIC categorization.

To accurately categorise PEUGIC, an adequate endoscopy needed to be defined. At present there is no definition of an adequate endoscopy and, therefore, the PEUGIC project team developed consensus criteria based on expert opinion. These are summarized in Table 2 .

**Table:** **Table 2** Criteria for adequate upper gastrointestinal endoscopy during the project.

Domain	Adequate	Inadequate
**Completeness**	Examination to D2 with J maneuver if possible Photo documentation of D2 and J maneuver Photo documentation of suspected malignant, cancer associated (e.g. gastric ulcer) or pre-malignant (e.g. Barrett’s esophagus) lesions	Unable to intubate the esophagus and D2
**Sampling**	When a suspected malignant lesion is found, at least 6 biopsies taken All gastric ulcers and severe esophagitis (LA grade D) should be biopsied adequately depending on size or have an adequate plan for repeat endoscopy and biopsy if this not possible on first endoscopy (e.g. due to bleeding)	Less than 6 biopsies from a suspected malignant lesion (exclusion lesions less than 1 cm in size)
**Equipment factors**	Absence of any of the criteria in the inadequate section of this domain	No image capture (with no documented reason for this)
**Patient factors**	Absence of any of the criteria in the inadequate section of this domain	Food residue preventing adequate mucosal views Bleeding preventing adequate mucosal views Poor patient tolerance including withdrawal of consent
**Report writing**	Follow-up endoscopy plan in line with BSG guidelines for cancer associated lesions (e.g. gastric ulcers)	Plan not in line with recommended BSG guidelines for follow-up
BSG, British Society of Gastroenterology. For endoscopy to be adequate, all criteria in the adequate section must be met, with no criteria in the inadequate section For surveillance or therapeutic endoscopy, the following modifications apply: The extent of the examination does not need to be to D2, provided there has been a proceeding endoscopy to D2 within the last 12 months. Biopsies for pre-malignant conditions such as Barrett's and gastric atrophy must be consistent with recommended protocols (Seattle and Sydney respectively). Surveillance or follow-up plan following histology results needs to be in line with BSG guidelines for surveillance of premalignant conditions or follow up of cancer associated lesions.		

### Data collection

Using data held by NDRS, PEUGIC diagnosed between January 1, 2017 and October 30, 2023 were identified. The following information was extracted: patient name, patient NHS number (all NHS patients have a unique code), patient date of birth, cancer diagnosis date, cancer diagnosis NHS provider, cancer diagnosis hospital, cancer morphology, cancer histology, index endoscopy date, index endoscopy NHS provider, and index endoscopy hospital.

Information about the most recent 25 PEUGIC by cancer diagnosis date was uploaded onto the “manage PEUGIC cases” dashboard of the portal for each provider. At the end of the data collection period, providers requested that all available PEUGIC were uploaded to the portal.

Reviewers were required to confirm patient eligibility before moving onto the PEUGIC root cause analysis. If a patient did not have a diagnosis of the relevant cancer or lacked an index endoscopy during the correct time period, reviewers were able to exclude the patient. The project team monitored reviewer justifications for excluded patients every 2 weeks and reinstated such patients to the portal if they had been inappropriately excluded, with email contact to explain the reasons for reinstatement.

When the cancer diagnosis and index endoscopy happened at different providers (20% of PEUGIC), the first section of the questionnaire was completed by the cancer diagnosis provider and, once completed, the questionnaire was automatically forwarded to the index endoscopy provider to review and complete the second half of the root cause analysis questionnaire.

### Data analysis

Data collected in the PEUGIC root cause analysis platform over 6 months and held by the NDRS were pseudonymized and sent to the PEUGIC project team for analysis.

As part of data cleaning, errors in PEUGIC categorization and endoscopy adequacy classification were corrected manually case by case. PEUGIC categorization was changed when not aligned with sections in the root cause analysis questionnaire on lesions detected and adequacy of index endoscopy. For endoscopies initially reported on review as adequate, later sections in the root cause analysis questionnaire if selected, such as absence of photo documentation or extent of the endoscopy not being the second part of the duodenum, rendered the endoscopy inadequate. Analysis was descriptive of the causes of PEUGIC and associated patient, procedure, and provider factors that contribute to its occurrence.

As part of the root cause analysis process, General Medical Council and Nursing and Midwifery Council numbers (professional registration details) were collected for the endoscopists who performed the index endoscopy. This was for later linkage of the PEUGIC root cause analysis dataset to the National Endoscopy Database (NED). NED contains key data for each endoscopy performed in the UK with near complete coverage of endoscopy providers 34 . Endoscopies performed by endoscopists with a PEUGIC identified in the root cause analysis project will be compared with endoscopists without a PEUGIC in the project, to identify differences in endoscopy practice between the two groups.

The research team estimated the time the root cause analysis questionnaire took to complete. This was measured as the time from the root cause analysis questionnaire being opened and viewed until submission of the questionnaire in minutes.

### Data protection and ethics considerations

NCRAS is responsible for population cancer registration of all patients diagnosed or treated in England with an invasive malignancy. NCRAS has been permitted to process patient-identifiable data without consent in accordance with the processing purposes outlined in Regulation 2, Health Services (Control of Patient Information) Regulations 2002. Access to patient-identifiable data within the organizational boundary of NHS England is controlled through appropriate organizational and technical measures (i.e. replacement of NHS numbers in the Cancer Analysis System with a patient ID to limit access to direct identifiers to a need-to-know basis). For the purpose of this study, NHS England acted as a data intermediary to identify and share clinical data about patients who have been registered by NCRAS as having a diagnosis of esophageal, gastric, or duodenal cancer.

NHS England in partnership with Health Data Insight Community Interest Company honorary contractors performed linkage of the NCRAS and HES datasets to identify patients with PEUGIC and distributed their data via the secure online portal to NHS endoscopy providers responsible for the patients in order for the providers to conduct the root cause analysis.

Health Data Insight has a data access contract with NHS England as a Partner Organization and completed the necessary Data Protection Impact Assessments, which were approved by the NDRS Caldicott Guardian. Following completion of each root cause analysis, the data were returned to NDRS for storage. In order for the research team to access and analyses the PEUGIC RCA dataset, a data access application was made to NHS England through NDRS. The pseudonymized dataset was then shared with the research team. No directly identifiable data were shared with the research team.

### Project management

#### Project management group

The Project Management Group consisted of the chief investigator, a lay representative, two members who previously led the national PCCRC root cause analysis project, representation from the Joint Advisory Group for Gastrointestinal Endoscopy, representation from the Association of Upper Gastrointestinal Surgeons, representation of clinical endoscopists and trainee endoscopists, along with Health Data Insight staff working on the project. All were responsible for the study and met every month during the planning and data collection period of the study and then quarterly following completion.

#### Ongoing project management and reviewer support

During the national rollout of the study, 3-monthly online question and answer sessions were run for reviewers and providers to drop in to ask the study team any questions or raise any issues. Presentations and advice relating to common queries, such as inappropriate exclusions, were created and disseminated during these sessions. In addition, an NHS email address was created for reviewers to email any queries related to the root cause analysis process and allowed direct contact between the study team and reviewers to facilitate PEUGIC analysis.

### Protocol version

The study is on protocol version 1.5 dated March 5, 2024.

## Results

### Study participation

A total of 3330 cases (85%) were reviewed of the total 3904 PEUGICs identified between January 1, 2017 and October 30, 2023. Root cause analysis was completed for 2666 (80%) PEUGICs and 664 (20%) were assessed as ineligible.

All 144 endoscopy providers in the English NHS were invited to take part in this study. Seven providers (5%) failed to submit a PEUGIC root cause analysis for the following reasons: one provider did not assign a project lead; one provider refused to take part due to unfounded information governance concerns; two experienced insurmountable information technology issues that prevented PEUGIC submission; and three failed to engage without providing reasons.

The provider PEUGIC case completion rate varied from 0% to 100% (median 100%; interquartile range [IQR] 87%-100%), with 76 of 144 providers (53%) completing all PEUGIC allocated to them. Five hundred and six PEUGIC (19%) had an index endoscopy at a different provider than the cancer diagnosis provider.

### Exclusions

One hundred fifty-three exclusions (23%) were excluded by local reviewers inappropriately. Common reasons for exclusion are summarized in Table 3 . Exclusions were divided into three main groups. Those where the cancer was distal to the second part of the duodenum or ampullary, where the case data were incorrect affecting eligibility for inclusion, or data were unavailable were excluded. Those where the case data were correct but local provider review suggested the case should be excluded were reviewed centrally by the project team and challenged if inappropriate by email with the local project team. The themes for exclusion when case data were correct but still excluded at local review included: missing or inaccessible documentation – original endoscopy report lost through transfer to a new electronic patient record; surgical factors affecting endoscopic assessment – eg previous Roux en Y procedure and cancer in excluded stomach or long-term impassible stricture; alternative pathologies – carcinoma of unknown primary or lymphoma; issues with the index procedure/system errors – reviewer believing the index endoscopy diagnosed cancer, but there was no histological confirmation; pre-cancerous conditions and progression – index endoscopy identified a nodule in a segment of Barrett’s with biopsies showing high-grade dysplasia, subsequent EMR histology showing early cancer; existing cancer diagnosis – previous esophageal squamous cell cancer treated with palliative radiotherapy (additional case exclusion examples are given in Supplementary Materials).

**Table:** **Table 3** Reasons for exclusion of potential PEUGIC at review in providers.

Main exclusion group	Percentage	Group themes	Percentage
Patient data were correct but case should be excluded	45%	Precancerous conditions and progression*	12.6%
		Missing or inaccessible documentation	11.9%
		Surgical factors affecting endoscopic assessment	7.0%
		Alternative pathologies	5.0%
		Issues with the index procedure/system errors*	25.6%
		Existing cancer diagnosis	4.0%
		No diagnosis of cancer	10.9%
		Multiple themes listed for exclusion	23.0%
Patient data were NOT correct or unavailable	45%	Cancer diagnosis date is incorrect	12.7%
		Cancer diagnosis hospital is incorrect	8.8%
		Cancer diagnosis provider is incorrect	17.8%
		Cancer histology is incorrect	1.8%
		Cancer site is incorrect	7.2%
		Index endoscopy date is incorrect	6.1%
		Index endoscopy hospital is incorrect	2.6%
		Index endoscopy provider is incorrect	7.7%
		NHS number is incorrect	0.4%
		No record of diagnosed cancer	9.9%
		No record of endoscopy performed	11.8%
		No record of patient	12.7%
		Patient name is incorrect	0.4%
Cancer site is Ampulla or D3 or D4 and therefore case should be excluded	10%		
*The majority of exclusions in the groups highlighted with an asterisk were inappropriate.			

Issues with the index procedure and pre-cancerous conditions and progression were the most commonly challenged because diagnosis of cancer requires histological confirmation and in many of these cases, reviewers were making an optical diagnosis.

### Time to complete root cause analysis questionnaire

**Fig. 3:**
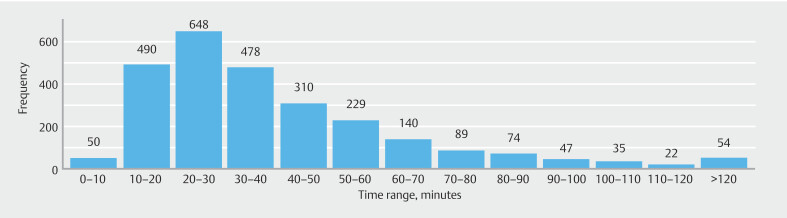
Histogram for the time taken to complete the root cause analysis questionnaire for each PEUGIC.

Median approximate time for PEUGIC review and completion of the root cause analysis questionnaire was 33 minutes (IQR 22–51). Fig. 3 shows histograms of the approximate time taken to complete the root cause analysis questionnaire.

### Data cleaning

Following data cleaning, PEUGIC assigned into category A decreased by 1.5%, in category B increased by 15%, in category C decreased by 15% and in category D increased by 4%. The number of index endoscopies classed as inadequate increased by 18%.

## Discussion

This study aimed to identify all PEUGIC in a national healthcare system and perform root cause analysis to identify causes and risk factors for PEUGIC. This study has generated the largest dataset of PEUGIC in the world and will provide valuable information on common causes of PEUGIC and how to reduce their incidence in the future. This study was based on the previous national PCCRC root cause analysis project, with the methodology adapted from that study. This methodology can be used in other areas of medicine where there is linkage between the date of a diagnostic test and diagnosis of cancer. For example, this methodology can be used to study cancers not detected in other endoscopic procedures or cancers not detected during radiological investigations. The granular detail provided by local root cause analysis provides much greater insight than population-level studies into PEUGIC and importantly, allows for targeted interventions on, for example, endoscopic practices associated with PEUGIC. Importantly we demonstrated the need of central quality control of PEUGIC RCA data to allow standardization through correction of local data entry to improve the validity of results.

### Strengths and limitations

For this study to be replicated, it requires a health service to have databases with registration of cancer diagnoses and registration of diagnostic tests such as endoscopy to identify potential PEUGIC. Ideally, these should be national datasets because up to 20% of PEUGIC are diagnosed at another organization and, therefore, local identification of PEUGIC may miss a considerable number of PEUGIC 10 . However, the root cause analysis questionnaire would allow endoscopy providers in other healthcare systems to use a standardized process to review the majority of their PEUGIC at a local level, even without national datasets. The process of completing a root cause analysis for each PEUGIC can be time-consuming and the approximate times recorded did not include the additional time needed for discussions concerning and actual discharge of the duty of candor (around 2% of cases in the national PCCRC root cause analysis project) 32 . Specific limitations of this study included the inability to include endoscopies that were performed at independent sector (non-NHS) providers or on patients who live on the border between England and the devolved nations of Wales and Scotland, when their endoscopies were performed outside England. Although central quality control enabled correction of classification errors in some PEUGIC, it was not possible to assess inter-assessor reliability.

## Conclusions

We have created a system that is able to identify diagnostic procedures that do not diagnose cancer in patients that go on to be diagnosed with cancer; in this case, for endoscopy. This methodology can be applied to other diagnostic tests in the future. We have also created a root cause analysis questionnaire, which allows for standardized review of PEUGIC, allowing immediate local learning. It has permitted national analysis of the largest cohort of PEUGIC in the world, to identify endoscopy practices associated with missed cancers and interventions to reduce their occurrence. This analysis will be the subject of future publications.

## Supplementary Material

Supplementary Material Zusatzmaterial
