# Supplementary material for: Development of a national root cause analysis system for post-endoscopy upper gastrointestinal cancer
Source: Endosc Int Open. 2026 Mar 16;14:a27883285. doi: 10.1055/a-2788-3285 (PMC13063305; doi:10.1055/a-2788-3285)
Supplement: Supplementary file 1 — Supplementary Material [file 10-1055-a-2788-3285_28155949.pdf]

Supplementary Table 1 ICD cancer morphology codes for upper gastrointestinal cancer as part of the criteria for PEUGIC.

ICD code	Description	ICD code	Description
150	Cervical esophagus	162	Body of stomach
151	Thoracic esophagus	163	Gastric antrum
152	Abdominal esophagus	164	Pylorus
153	Upper third of esophagus	165	Lesser curvature of stomach
154	Middle third of esophagus	166	Greater curvature of stomach
155	Lower third of esophagus	167	Gastroesophageal junction
158	Overlapping lesion of esophagus	168	Overlapping lesion of stomach
159	Esophagus, not specified	169	Stomach, not specified
160	Cardia of stomach	170	Duodenum
161	Fundus of stomach		

ICD, International Classification of Diseases.

Supplementary Table 2 ICD cancer histology codes for upper gastrointestinal cancer as part of the criteria for PEUGIC.

ICD code	Description	ICD code	Description
8010	Carcinoma	8210	Adenocarcinoma in adenomatous polyp
8020	Carcinoma, undifferentiated	8211	Tubular adenocarcinoma
8021	Carcinoma, anaplastic	8213	Serrated adenocarcinoma
8022	Pleomorphic carcinoma	8214	Parietal cell carcinoma
8032	Spindle cell carcinoma	8231	Carcinoma simplex
8033	Pseudosarcomatous carcinoma	8245	Adenocarcinoid tumor
8041	Small cell carcinoma	8255	Adenocarcinoma with mixed subtypes
8043	Small cell carcinoma, fusiform cell	8260	Papillary adenocarcinoma
8044	Small cell carcinoma, intermediate cell	8261	Adenocarcinoma in villous adenoma
8045	Combined small cell carcinoma	8262	Villous adenocarcinoma
8050	Papillary carcinoma	8263	Adenocarcinoma in tubulovillous adenoma
8051	Verrucous carcinoma	8310	Clear cell adenocarcinoma
8052	Papillary squamous cell carcinoma	8323	Mixed cell adenocarcinoma
8070	Squamous cell carcinoma	8430	Mucoepidermoid carcinoma
8071	Squamous cell carcinoma, keratinizing	8440	Cystadenocarcinoma
8072	Squamous cell carcinoma, large cell, nonkeratinizing	8480	Mucinous adenocarcinoma
8073	Squamous cell carcinoma, small cell, nonkeratinizing	8481	Mucin-producing adenocarcinoma
8074	Squamous cell carcinoma, spindle cell	8490	Signet ring cell carcinoma
8075	Squamous cell carcinoma, adenoid	8510	Medullary carcinoma
8076	Squamous cell carcinoma, microinvasive	8512	Medullary carcinoma with lymphoid stroma
8078	Squamous cell carcinoma with horn formation	8560	Adenosquamous carcinoma
8083	Basaloid squamous cell carcinoma	8562	Epithelial-myoepithelial carcinoma
8084	Squamous cell carcinoma, clear cell type	8570	Adenocarcinoma with squamous metaplasia

8140	Adenocarcinoma	8571	Adenocarcinoma with cartilaginous and osseous metaplasia
8141	Scirrhous adenocarcinoma	8573	Adenocarcinoma with apocrine metaplasia
8142	Linitis plastica	8574	Adenocarcinoma with neuroendocrine differentiation
8143	Superficial spreading adenocarcinoma	8575	Metaplastic carcinoma
8144	Adenocarcinoma, intestinal type	8576	Hepatoid adenocarcinoma
8145	Carcinoma, diffuse type	8980	Carcinosarcoma
8190	Trabecular adenocarcinoma	8982	Malignant myoepithelioma
8200	Adenoid cystic carcinoma	9100	Choriocarcinoma
8201	Cribriform carcinoma	8000	Neoplasm, malignant

ICD, International Classification of Diseases.

Supplementary Table 3 OPSC4 procedure codes for endoscopy as part of the criteria for PEUGIC.

OPCS4 code	Description	OPCS4 code	Description
121	Fiberoptic endoscopic mucosal resection of lesion of esophagus	432	Fiberoptic endoscopic laser destruction of lesion of upper gastrointestinal tract
128	Other specified other fiberoptic endoscopic extirpation of lesion of esophagus	433	Fiberoptic endoscopic cauterization of lesion of upper gastrointestinal tract
141	Fiberoptic endoscopic snare resection of lesion of esophagus	434	Fiberoptic endoscopic sclerotherapy to lesion of upper gastrointestinal tract
142	Fiberoptic endoscopic laser destruction of lesion of esophagus	435	Fiberoptic endoscopic destruction of lesion of upper gastrointestinal tract NEC
143	Fiberoptic endoscopic cauterization of lesion of esophagus	436	Fiberoptic endoscopic injection therapy to lesion of upper gastrointestinal tract NEC
144	Fiberoptic endoscopic injection sclerotherapy to varices of esophagus	437	Fiberoptic endoscopic rubber band ligation of upper gastrointestinal tract varices
145	Fiberoptic endoscopic destruction of lesion of esophagus NEC	438	Other specified fiberoptic endoscopic extirpation of lesion of upper gastrointestinal tract
146	Fiberoptic endoscopic submucosal resection of lesion of esophagus	439	Unspecified fiberoptic endoscopic extirpation of lesion of upper gastrointestinal tract
147	Fiberoptic endoscopic photodynamic therapy of lesion of esophagus	44	Other therapeutic fiberoptic endoscopic operations on upper gastrointestinal tract
148	Other specified fiberoptic endoscopic extirpation of lesion of esophagus	441	Fiberoptic endoscopic insertion of prosthesis into upper gastrointestinal tract
149	Unspecified fiberoptic endoscopic extirpation of lesion of esophagus	442	Fiberoptic endoscopic removal of foreign body from upper gastrointestinal tract
151	Fiberoptic endoscopic removal of foreign body from esophagus	443	Fiberoptic endoscopic dilation of upper gastrointestinal tract NEC
152	Fiberoptic endoscopic balloon dilation of esophagus	445	Fiberoptic endoscopic percutaneous insertion of gastrostomy
153	Fiberoptic endoscopic dilation of esophagus NEC	446	Fiberoptic endoscopic pressure-controlled balloon dilation of lower esophageal sphincter

154	Fiberoptic endoscopic insertion of tubal prosthesis into esophagus	447	Fiberoptic endoscopic removal of gastrostomy tube
155	Fiberoptic endoscopic dilation of web of esophagus	448	Other specified other therapeutic fiberoptic endoscopic operations on upper gastrointestinal tract
156	Fiberoptic endoscopic insertion of expanding metal stent into esophagus NEC	449	Unspecified other therapeutic fiberoptic endoscopic operations on upper gastrointestinal tract
157	Fiberoptic endoscopic insertion of expanding covered metal stent into esophagus	451	Fiberoptic endoscopic examination of upper gastrointestinal tract and biopsy of lesion of upper gastrointestinal tract
158	Other specified other therapeutic fiberoptic endoscopic operations on esophagus	452	Fiberoptic endoscopic ultrasound examination of upper gastrointestinal tract
159	Unspecified other therapeutic fiberoptic endoscopic operations on esophagus	453	Fiberoptic endoscopic insertion of Bravo pH capsule into upper gastrointestinal tract
161	Diagnostic fiberoptic endoscopic examination of esophagus and biopsy of lesion of esophagus	454	Fiberoptic endoscopic examination of upper gastrointestinal tract and staining of gastric mucosa
168	Other specified diagnostic fiberoptic endoscopic examination of esophagus	458	Other specified diagnostic fiberoptic endoscopic examination of upper gastrointestinal tract
169	Unspecified diagnostic fiberoptic endoscopic examination of esophagus	459	Unspecified diagnostic fiberoptic endoscopic examination of upper gastrointestinal tract
20	Therapeutic fiberoptic endoscopic operations on esophagus	462	Fiberoptic endoscopic coagulation of bleeding lesion - upper gastrointestinal tract
201	Fiberoptic endoscopic coagulation of bleeding lesion of esophagus	468	Other specified therapeutic fiberoptic endoscopic operations on upper gastrointestinal tract
208	Other specified therapeutic fiberoptic endoscopic operations on esophagus	469	Unspecified therapeutic fiberoptic endoscopic operations on upper gastrointestinal tract
209	Unspecified therapeutic fiberoptic endoscopic operations on esophagus	541	Endoscopic extirpation of lesion of duodenum
215	Insertion of stent into esophagus NEC	542	Endoscopic dilation of duodenum
420	Other fiberoptic endoscopic extirpation of lesion of upper gastrointestinal tract	543	Endoscopic insertion of tubal prosthesis into duodenum
421	Fiberoptic endoscopic submucosal resection of lesion of upper gastrointestinal tract	548	Other specified therapeutic endoscopic operations on duodenum

422	Fiberoptic endoscopic photodynamic therapy of lesion of upper gastrointestinal tract	549	Unspecified therapeutic endoscopic operations on duodenum
423	Fiberoptic endoscopic mucosal resection of lesion of upper gastrointestinal tract	551	Diagnostic endoscopic examination of duodenum and biopsy of lesion of duodenum
428	Other specified other fiberoptic endoscopic extirpation of lesion of upper gastrointestinal tract	558	Other specified diagnostic endoscopic examination of duodenum
429	Unspecified other fiberoptic endoscopic extirpation of lesion of upper gastrointestinal tract	559	Unspecified diagnostic endoscopic examination of duodenum
431	Fiberoptic endoscopic snare resection of lesion of upper gastrointestinal tract		

NEC, neuroendocrine carcinoma; OPCS, Office of Population Censuses and Surveys Classification of Interventions and Procedures version 4.

Themes for exclusion where there was no case data error and examples

1. Missing or inaccessible documentation
"Had index endoscopy at another hospital."
"Cannot see an index endoscopy."
"No index / previous gastroscopy was done in our Trust. Only one gastroscopy was the procedure that showed cancer."

2. Anatomical/Surgical factors affecting endoscopic assessment
"Long term stricture due to esophageal pemphigoid with multiple dilatations since xxxx."

"Patient had roux en y procedure in past. OGD - xx/xx/xxxx - NAD. CT showed primary likely to be in excluded stomach (not visible at OGD) with metastatic disease."

3. Diagnostic ambiguity / alternative pathologies
"This was a carcinoma of unknown primary - it was never clear that it originated in the esophagus and no lesion was ever seen at endoscopy or on CT in the esophagus, however biopsies from metastatic bone lesions suggested an adenocarcinoma of lung/esophageal origin."
"Histology results following intraoperative OGD confirmed Marginal zone lymphoma."
"The cancer which was found in the stomach was consistent with the metastatic disease from the primary renal cell carcinoma."

4. Issues with the index procedure and system errors
These exclusions were often challenged as the index endoscopy must lead to a histological or radiological diagnosis of cancer and cannot be an endoscopists endoscopic diagnosis alone.
"Index endoscopy itself has diagnosed the lesion."
"This patient had Squamous cell carcinoma in situ diagnosed at the index endoscopy. I think why it is triggering on the system is that there was a system error where the histology was sent to the GP rather than the consultant. Therefore delay in diagnosis."
"Cancer was found at this index gastroscopy."
"The initial endoscopy … not well tolerated. cancer was detected on the second scope."

5. Pre-cancerous conditions and progression
These exclusions were often challenges as despite in most circumstances would class an good care the definition of PEUGIC is strict. If the preceding endoscopy did not show cancer even if it was high grade dysplasia which lead to a successful endoscopic resection if the time window between the 2 endoscopies was over 3 months it would still be classed as a PEUGIC.
"Index OGD xx identified esophageal high grade dysplasia in the context of Barrett's esophagus... The xx OGD showed a focal nodule... This changed to cancer during the long delay..."
"EMR of Barrett's final report: carcinoma in situ."
“Index endoscopy was for UGIB with bleeding GU not biopsied. Diagnosis made on follow-up”

6. Historical diagnoses/non-new cancer
"History of distal esophageal SCC diagnosed xxxx - for palliative intent with radiotherapy. To exclude as not a new cancer (xxxx histology has shown biopsies of SCC in situ in the same location)."
